# Three Cases of Human Babesiosis, Italy, 2017–2020

**DOI:** 10.3201/eid3106.241776

**Published:** 2025-06

**Authors:** Chiara Sepulcri, Rachele Pincino, Federico Baldi, Giovanni Cenderello, Stefania Zanet, Daniela Boccolini, Anna Rosa Sannella, Mariangela L’Episcopia, Carlo Severini, Matteo Bassetti, Chiara Dentone, Ezio Ferroglio

**Affiliations:** University of Genoa, Genoa, Italy (C. Sepulcri, M. Bassetti); Ospedale Sanremo, Sanremo, Italy (R. Pincino, G. Cenderello); AON SS Antonio e Biagio e Cesare Arrigo University Hospital, Alessandria, Italy (F. Baldi); University of Turin, Turin, Italy (S. Zanet, E. Ferroglio); Istituto Superiore di Sanità, Rome, Italy (D. Boccolini, A.R. Sannella, M. L’Episcopia, C. Severini); IRCCS Ospedale Policlinico San Martino, Genoa (M. Bassetti, C. Dentone)

**Keywords:** babesiosis, Babesia, piroplasmosis, One Health, arthropod-borne diseases, ticks, parasites, tick-borne, vector-borne infections, zoonoses, Italy

## Abstract

We report 3 cases of babesiosis in Italy caused by *Babesia* species that are rarely reported in humans. The circulation of *Babesia* spp. among vectors, animals, and humans might be more common than previously thought, and babesiosis might be an underdiagnosed and emerging disease in Italy and Europe.

Babesiosis is a tickborne disease caused by apicomplexan hemoprotozoan parasites of the genus *Babesia*. The biologic cycle of *Babesia* spp. involves species-specific interactions among the pathogen, host, and reservoir (*1*). More than 100 *Babesia* spp. have been described as cause of disease in animals, but only a few are involved in human infections.

Most human babesiosis cases have been reported in North America, where *B. microti* is the main pathogen (*1*). In Europe, where most infections are caused by either *B. divergens* or *B. venatorum* (*2*), only ≈50 human cases have been reported since 1957 (*3*). In Italy, only 1 case has been reported, in 2004 (*4*). In contrast to the paucity of clinically relevant cases, results from a seroprevalence study in Spain suggest that human infection might be more common; seroreactivity up to 39.2% in tick-exposed persons has been reported (*5*).

The growing use of molecular techniques is enabling new insights into the epidemiology of *Babesia* spp. in wildlife and domestic animals (*6*), as well as enabling identification of species that were previously unreported in humans, such as *B. bovis* and *B. canis* (*7*). We describe 3 clinical cases of human babesiosis that occurred in a northern region of Italy. All patients provided written consent for the use of their medical records for scientific research purposes at the time of hospital admission according to local legislation.

## The Study

To molecularly identify *Babesia* and *Theileria* spp. parasites, we extracted total genomic DNA from 200 µL of whole blood in EDTA by using the GenElute Mammalian Genomic DNA Miniprep Kit (Sigma-Aldrich, https://www.sigmaaldrich.com) according to the manufacturer’s instructions. We performed direct molecular detection of *Babesia* and *Theileria* spp. DNA by using a seminested PCR protocol targeting a 400-bp fragment of the V4 hypervariable region of the 18S ribosomal RNA gene, as previously described (*6*). We purified PCR positive samples by using the QIAquick PCR Purification Kit (QIAGEN, https://www.qiagen.com). Macrogen (http://www.macrogen.com) directly sequenced both DNA strands. We compared the sequences with those in Genbank by using BLAST (https://blast.ncbi.nlm.nih.gov) and used ClustalX software (http://www.clustal.org) to construct multiple sequence alignments. We analyzed the phylogenetic relationships of *Babesia* spp. isolates by using the maximum-likelihood method and the Jukes-Cantor model with 1,000 bootstrap replicates. We performed nested PCR for *Plasmodium* spp. detection, as previously described (*8*).

**Figure 1:**
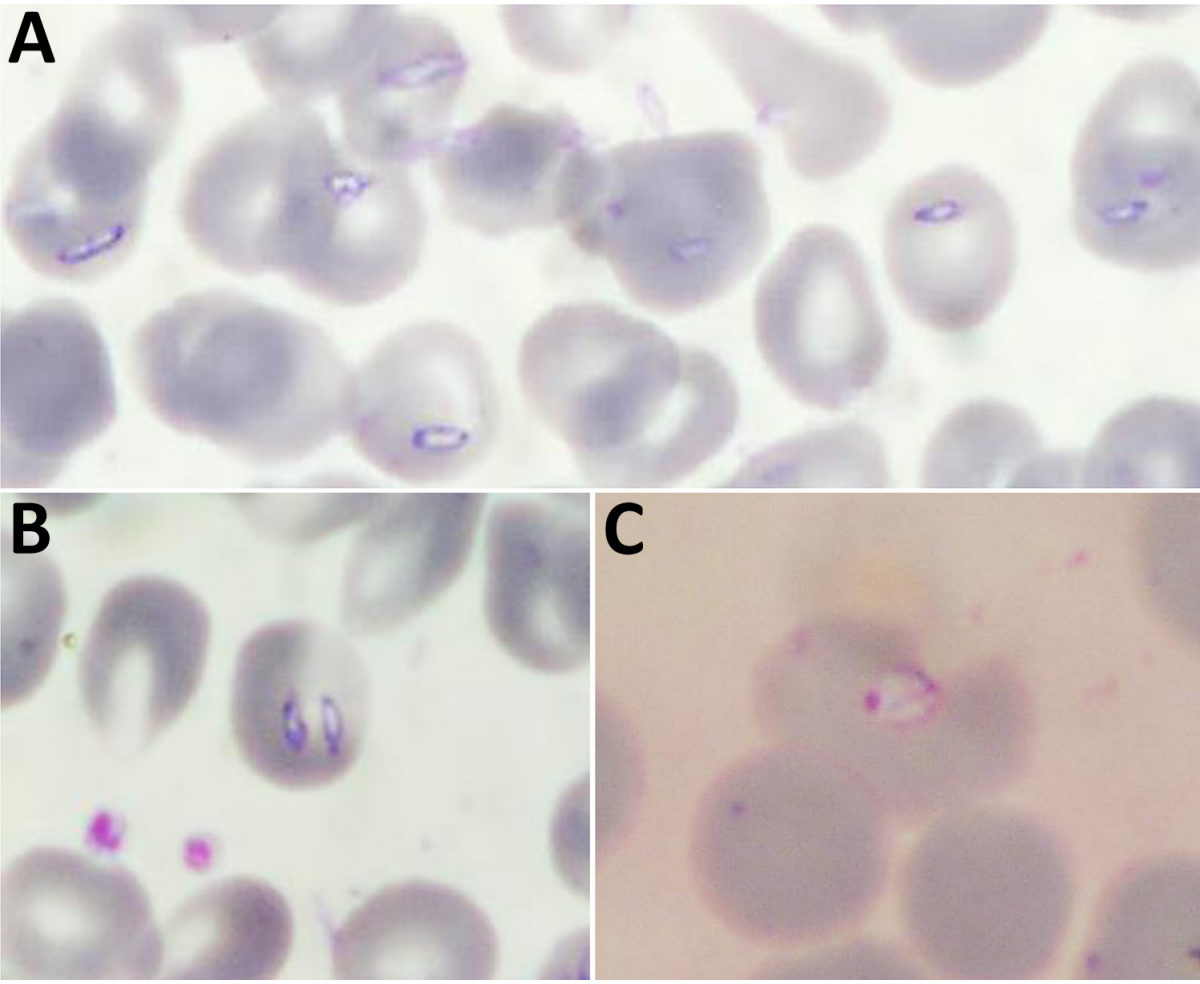
Blood smears showing *Babesia* spp. trophozoites in study of 3 cases of human babesiosis, Italy, 2017–2020. Smears were stained with Giemsa stain. A, B) *B. canis canis* identified in patient 1. Original magnification ×630. C) *B. vulpes* identified in patient 3. Original magnification ×1,000 (oil immersion).

**Figure 2:**
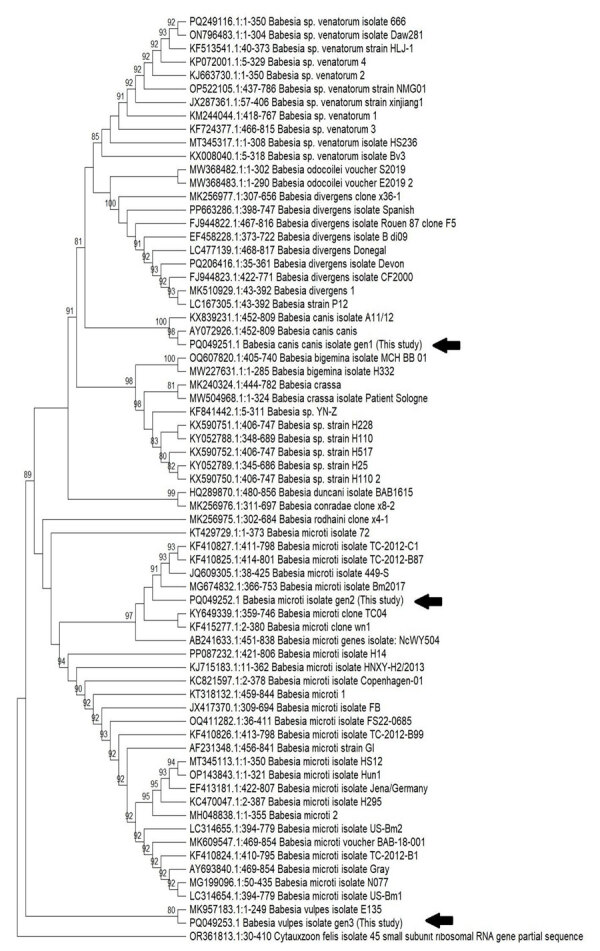
Phylogenetic analysis of the *Babesia* spp. in study of 3 cases of human babesiosis, Italy, 2017–2020. 18S ribosomal RNA gene sequences were amplified from the 3 clinical isolates from Italy. Arrows indicate the *Babesia* isolates from the 3 patients. Tree was inferred by using the maximum-likelihood method with 1,000 bootstrap replicates. Tree with the highest log likelihood (−3,487.14) is shown. Bootstrap values (cutoff at 80%) are indicated at specific branch nodes. 18S ribosomal RNA gene (partial) from *Cytauxzoon felis* protozoan parasite was used as the outlier. Tree not drawn to scale.

Patient 1 was a woman in her 20s with a history of Raynaud syndrome. In 2017, she was admitted to a hospital in the western part of northern Italy for arthralgia, myalgia, asthenia, fever, and weight loss, all occurring during the previous 2 weeks. She lived with 2 dogs and had no history of recent travel or transfusions with any blood components. Her clinical evaluation and bloodwork were unremarkable. She had negative results for HIV, hepatitis C virus, *Borrelia* and *Bartonella* spp. antibodies, autoimmunity tests, and blood and urine cultures. To rule out hematologic disorders, we prepared blood smears and found evidence of trophozoite forms. A malaria rapid test was negative for all *Plasmodium* spp. The National Reference Laboratory in Rome, Italy, performed microscopic examination of the smears, which showed both extraerythrocytic and intraerythrocytic trophozoites, suggesting *Babesia* spp. (Figure 1, panels A, B). The local Zooprofilactic Institute confirmed that finding by performing *Babesia* spp. PCR 40 days after the patient’s hospital admission. Further molecular analysis identified the species as *B. canis canis* (Genbank accession no. PQ049251; 100% query coverage and maximum identity) (Figure 2). Treatment consisted of a 10-day course of oral atovaquone (750 mg 2×/d) and azithromycin (500 mg 1×/d), which led to symptom remission and a negative blood smear. One of the patient’s 2 dogs was positive for *B. canis canis* by species-specific seminested PCR, conducted as previously described (*9*).

Patient 2 was a child <10 years of age admitted in 2018 to the same hospital as patient 1 for headache, arthralgia, myalgia, pharyngitis with tonsillitis, and fever that began 8 days before admittance. The patient had no travel history or history of transfusions with blood components. Blood exams revealed hypereosinophilia (24,200 cells/μL). Blood microscopy showed the presence of scant trophozoites; a malaria rapid diagnostic test and PCR were negative for all *Plasmodium* spp. Suspecting babesiosis, we sent a blood sample to the local Zooprofilactic Institute to test for *Babesia* spp. by PCR 2 days after admission; results were positive for babesiosis, and *B. microti*–like species was identified (Genbank accession no. PQ049252; 100% query coverage, 95.36% maximum identity) (Figure 2). Treatment consisted of a 7-day course of intravenous azithromycin (10 mg/kg 1×/d) and atovaquone (20 mg/kg 2×/d). Symptoms resolved (afebrile, resolution of arthralgia and myalgia) after 3 days of treatment, and a negative blood smear was observed.

Patient 3 was a man in his 20s born in West Africa admitted in 2020 for fever, night sweats, and productive cough that began 1 month before admittance. The patient had been admitted the year before to the same pulmonary tuberculosis (TB) unit but was subsequently lost to follow-up. At admission, he was stably residing in Italy and had not traveled abroad in the previous 5 years. We diagnosed pulmonary TB and started treatment with rifampin, isoniazid, ethambutol, and pyrazinamide. At the time of admission, he had fever, anemia (hemoglobin level 6.6 g/dL), hemoglobinuria, and elevated inflammatory markers. Three weeks after admission, he had persistent fever and anemia despite TB treatment and blood transfusions. We suspected malaria reactivation and prepared thick and thin blood smears, which showed intraerythrocytic trophozoites. The National Reference Laboratory performed a rapid malaria test and nested PCR, which were negative for all *Plasmodium* spp. We suspected babesiosis, which we confirmed by microscopy of a blood smear (Figure 1, panel C). Parasitemia was 0.2%. Further molecular testing identified the species as *B. vulpes* (Genbank accession no. PQ049253; 71% query coverage, 97.29% maximum identity) (Figure 2). We treated the patient with oral quinine and intravenous clindamycin. After 10 days of therapy, he was still symptomatic with persistent parasitemia observed in blood smears. We extended the treatment duration to 21 days, resulting in symptoms resolution and repeated negative blood smears.

## Conclusions

The 3 human babesiosis cases described here were sustained by *Babesia* spp. rarely reported as a cause of human disease. Although none of the patients specifically reported a history of tick bites, all had potential environmental exposure to ticks. The circulation of *Babesia* spp. in Italy has been increasingly recognized both in its vector (mainly *Ixodes ricinus* ticks) (*10*) and in wild and domestic animals (*11*). In particular, *B. vulpes* was recently isolated from wild boars (*Sus scrofa*) in southern Italy (*12*). The population of wild boars in Italy is expanding and, in Genoa, where the case of *B. vulpes* was reported, the presence of wild boars within an urban space is an increasingly critical phenomenon (*13*). Furthermore, as shown by a seroprevalence study conducted in central and northern Italy, seroreactivity to *Babesia* spp. was detected in 24.4% of persons with high tick exposure (i.e., foresters, hunters) and 7% in persons with a lower exposure risk (*14*), suggesting a high rate of infection in the population. Current ecologic changes are likely to influence the emergence and prevalence of zoonotic diseases worldwide; the complex interactions among climate, pathogens, vectors, and hosts are dynamically changing and warrant the implementation of a One Health approach (*15*). Babesiosis is an example of those complex interactions and might be an underdiagnosed and emerging disease in Italy and Europe.
